# MUC1-C drives DNA methylation in cancer

**DOI:** 10.18632/aging.101153

**Published:** 2016-12-28

**Authors:** Hasan Rajabi, Ashujit Tagde, Donald Kufe

**Affiliations:** Dana-Farber Cancer Institute, Harvard Medical School, Boston, MA 02215, USA

**Keywords:** MUC1-C, DNMT, DNA methylation, tumor suppressor genes, EMT

Recent publications have reported a previously unrecognized role for the MUC1-C oncoprotein in regulating DNA methyltransferase (DNMT) expression and thereby DNA methylation in human cancer cells [1, 2]. The MUC1-C transmembrane protein is aberrantly overexpressed in diverse human cancers and in certain hematologic malignancies, including acute myelo-genous leukemia (AML) [3]. Dysregulation of DNMTs and disruption of DNA methylation patterns are established hallmarks of the cancer cell [4]. MUC1-C has been linked to other hallmarks, such as (i) induction of the epithelial-mesenchymal transition (EMT), (ii) repression of tumor suppressor genes, (iii) activation of the *MYC* gene, and (iv) promotion of self-renewal capacity [3, 5]. However, there had been no known relationship between MUC1-C-induced signaling and the regulation of DNMTs and DNA methylation in cancer.

DNMTs catalyze the transfer of a methyl group to cytosine in CpG dinucleotides [4]. DNMT1, which localizes at foci of DNA replication, is largely responsible for maintaining global and gene-specific CpG methylation. In addition, DNMT3a and DNMT3b typically contribute to *de novo* postreplicative DNA methylation patterns. DNA methylation in promoter regions is associated with transcriptional repression; whereas, DNA methylation in gene bodies can induce increases in transcription and alternative splicing [4]. In this respect, dysregulation of DNMTs in cancer cells can affect global programs of gene repression and activation. Despite this complexity, a notable finding is that both DNMT1 and DNMT3b are required for silencing genes in cancer cells [6].

**Figure 1:**
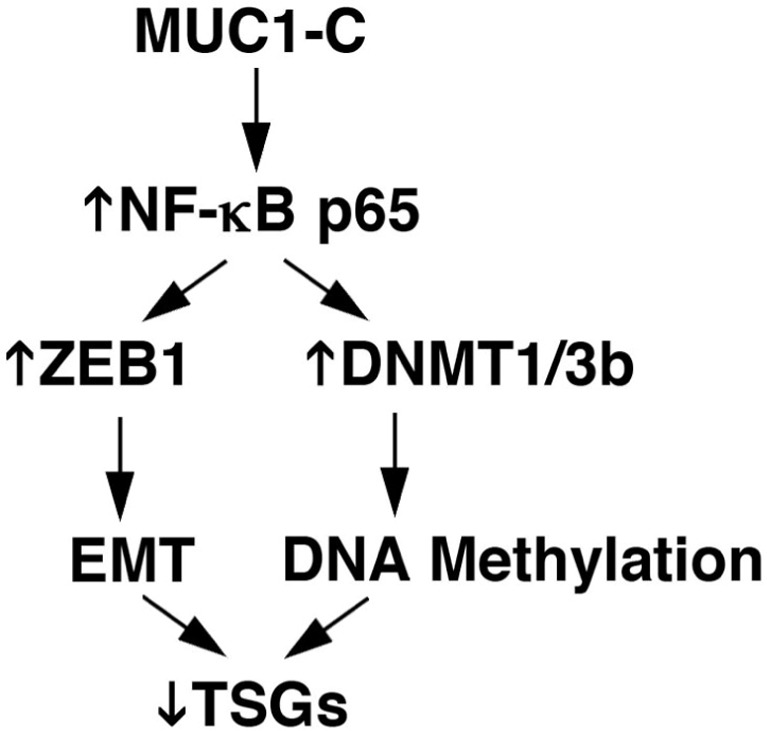
MUC1-C→NF-κB signaling integrates EMT and DNA methylation-induced changes in gene expression MUC1-C activates the inflammatory TAK1→IKK→NF-κB p65 pathway. MUC1-C also binds directly to NF-κB p65, increases occupancy of MUC1-C/NF-κB p65 complexes on the *ZEB1* promoter, induces ZEB1 expression, and drives EMT by ZEB1-mediated mechanisms. Additionally, MUC1-C increases MUC1-C/NF-κB p65 occupancy on the *DNMT1/3b* promoters, induces DNMT1/3b expression, and alters global and gene-specific DNA methylation patterns. In this way, MUC1-C→NF-κB p65 signaling plays a role in integrating EMT with epigenetic changes in gene expression and the repression of TSGs.

The MUC1-C cytoplasmic domain is a 72-amino acid intrinsically disordered protein, a finding consistent with other oncogenic molecules that have the plasticity to direct the activation of multiple signaling pathways. Along these lines, the MUC1-C cytoplasmic domain interacts with diverse kinases and effectors that are linked to transformation [3, 5]. For instance, the MUC1-C cytoplasmic activates the WNT pathway by binding directly to β-catenin and promoting the activation of WNT target genes, such as *CCND1* and *MYC.* MUC1-C also activates the inflammatory IKK→NF-κB pathway and drives the expression of NF-κB p65 target genes, including *MUC1* itself in an auto-inductive loop. In addition, the MUC1-C→NF-κB p65 pathway induces the *ZEB1* tumor suppressor gene and promotes ZEB1-mediated repression of genes encoding (i) factors, such as Crumbs 3 (CRB3), required for maintaining apical-basal polarity, (ii) miR-200c, an inducer of epithelial differentiation, and (iii) E-cadherin, a keystone of the adherens junction complex; all of which contribute to the EMT phenotype (Fig. 1) [7].

In concert with driving EMT, recent work has linked the MUC1-C→NF-κB pathway to the regulation of DNA methylation in carcinoma cells (Fig. 1) [1]. Interestingly, the findings demonstrate that MUC1-C induces the expression of DNMT1 and DNMT3b, but not DNMT3a [1], in line with the requirement for both DNMT1 and DNMT3b to silence genes in cancer cells [6]. MUC1-C drives NF-κB p65 occupancy on the *DNMT1* and *DNMT3b* promoters and promotes their activation [1]. In this way, MUC1-C regulates global DNA methylation and induces *CDH1* promoter methylation with suppression of E-cadherin expression. These findings and the role of MUC1-C in inducing EMT have invoked the likelihood that MUC1-C epigenetically regulates other genes in the EMT program (Fig. 1) [7]. In addition and as raised below, these findings support the notion that MUC1-C is a potential target for derepressing tumor suppressor genes (TSGs), such as *CDH1* and others.

DNMT1 is necessary for the self-renewal of leukemia stem cells. Moreover, the anti-leukemic agent decita-bine downregulates DNMT1, but has no effect on DNMT3a/b expression, supporting the potential importance of DNMT1 as a target for the treatment of AML [2]. In this regard and based on the results obtained in carcinoma cells, MUC1-C was found to induce DNMT1 expression by an NF-κB p65-dependent mechanism in AML cells [2]. Targeting MUC1-C with silencing or pharmacologically with the inhibitor GO-203 in AML cell lines and primary blasts was thus associated with (i) the suppression of DNMT1, (ii) decreases in methylation of CpG islands in the *CDH1* promoter, and (iii) upregulation of E-cadherin [2]. Targeting MUC1-C in AML cells was also associated with derepression of the *PTEN* and *BRCA1* TSGs [2].

The available evidence thus supports an important role for MUC1-C in the dysregulation of DNA methylation in human cancer cells [1, 2]. Therefore, targeting MUC1-C could represent a therapeutic approach alone and in combination with agents, such as decitabine, for reprogramming the cancer epigenome. Indeed, the combination of GO-203 and decitabine has been shown to be highly effective in downregulating DNMT1 and decreasing AML cell survival [2]. With regard to the potential for clinical translation, GO-203 has completed Phase I evaluation in patients with advanced carcinomas. Moreover, based on the above findings, a Phase Ib/IIa trial of GO-203 in combination with decitabine is underway for the treatment of patients with relapsed/refractory AML.
